# Synergies of Systems Biology and Synthetic Biology in Human Microbiome Studies

**DOI:** 10.3389/fmicb.2021.681982

**Published:** 2021-08-31

**Authors:** Bouchra Ezzamouri, Saeed Shoaie, Rodrigo Ledesma-Amaro

**Affiliations:** ^1^Unit for Population-Based Dermatology Research, St John’s Institute of Dermatology, Guy’s and St Thomas’ NHS Foundation Trust and King’s College London, London, United Kindom; ^2^Faculty of Dentistry, Centre for Host-Microbiome Interactions, Oral and Craniofacial Sciences, King’s College London, London, United Kingdom; ^3^Department of Bioengineering and Imperial College Centre for Synthetic Biology, Imperial College London, London, United Kingdom; ^4^Science for Life Laboratory, KTH—Royal Institute of Technology, Stockholm, Sweden

**Keywords:** microbiome, synthetic biology, systems biology, microbioime engineering, microbiome therapies

## Abstract

A number of studies have shown that the microbial communities of the human body are integral for the maintenance of human health. Advances in next-generation sequencing have enabled rapid and large-scale quantification of the composition of microbial communities in health and disease. Microorganisms mediate diverse host responses including metabolic pathways and immune responses. Using a system biology approach to further understand the underlying alterations of the microbiota in physiological and pathological states can help reveal potential novel therapeutic and diagnostic interventions within the field of synthetic biology. Tools such as biosensors, memory arrays, and engineered bacteria can rewire the microbiome environment. In this article, we review the computational tools used to study microbiome communities and the current limitations of these methods. We evaluate how genome-scale metabolic models (GEMs) can advance our understanding of the microbe–microbe and microbe–host interactions. Moreover, we present how synergies between these system biology approaches and synthetic biology can be harnessed in human microbiome studies to improve future therapeutics and diagnostics and highlight important knowledge gaps for future research in these rapidly evolving fields.

## Introduction

**FIGURE 1:**
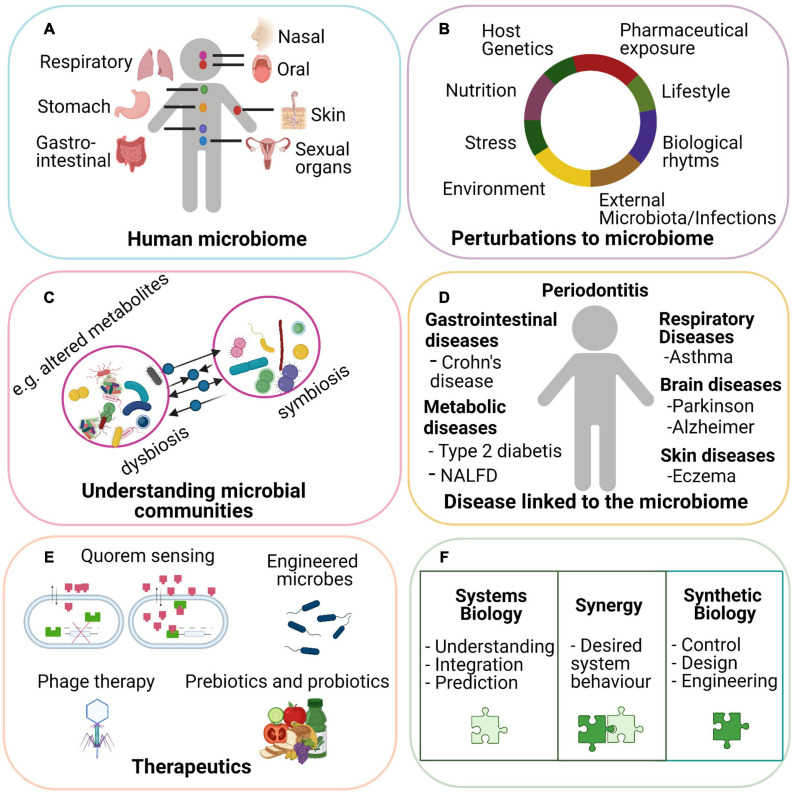
Microbiota is involved in normal host physiology and can be a contributing cause to many diseases. Microbial communities are present at different body sites in the human body **(A)**. An individual’s microbiota can be influenced by several internal and external factors such as age diet, genetics, and medication **(B)**. When the homeostatic balance of the community is disrupted, a dysbiosis can occur leading to altered or new exchanged metabolites, pathways, or mechanisms **(C)**. A persistent change in symbiotic and dysbiotic microbial communities can have a preventive or promotor role in the development of several diseases **(D)**. With the help of synthetic biology, the host equilibrium can be restored with effective therapeutics such as engineering microbes to detect external signal and integrating these inputs to deliver therapeutics. Phage therapy relies on recognizing a target bacterial species engineered by phage particles so that specific species with certain genes can be eliminated. Administrated targeted probiotics and prebiotics can stimulate the growth of certain bacterial species in which a community can be restored. Quorum sensing involves cell-to-cell communication mediated by diffusible signal molecules and can be used for development of personalized and translational medicine. Synthetic biology can add to the field of systems biology by developing effective treatment and diagnostic strategies **(E)**. Synergies of systems biology and synthetic biology in human microbiome studies are vital for a better understanding of microbial communities in health and disease **(F)**.

The human microbiota consist of microorganisms, including bacteria, viruses, and fungi, that live in and on the human body (Ursell et al., 2012; Figure 1A). The composition of these microbial communities varies with body site (Ma et al., 2018) and can be influenced by several factors such as age, diet, drugs, and sex (Hollister et al., 2014; Figure 1B). The human microbiota play various roles in physiological functions including development of the immune system (Mezouar et al., 2018), drug metabolism (Nichols et al., 2019), nutrient degradation, protection against pathogens, and vitamin production (Rowland et al., 2018). Moreover, studies have shown that alterations of the homeostatic balance of gut microbial communities (dysbiosis) can be associated with disease including infectious diseases (e.g., *Clostridium difficile*; Bien et al., 2013), metabolic diseases (Karlsson et al., 2013; Bouter et al., 2017), and inflammatory bowel disease (Tamboli, 2004; Figures 1C,D).

Microbial communities are dynamic, and members of the community fluctuate over time resulting in changes in overall microbial diversity (Figure 1D). Understanding and controlling microbial communities can help maintain health and treat disease by restoring host–microbiota homeostasis. There are complex interactions between the microbiome and host as well as microbe–microbe interactions and therefore, a systems-level approach is needed to better understand these interactions and describe the microbiome changes underlying mechanisms of health and disease. Systems biology approaches aim to describe complex cellular and/or tissue interactions by implementing biological networks and using mathematical models (Breitling, 2010). Moreover, the modeling of biological networks can function as a tool for the integration and exploration of multi-omics data to create a more holistic understanding of microbial communities. Integration of our current knowledge of systems biology methods with the field of synthetic biology provides an approach to manipulate and design microbial systems in which human health and treatment of disease can be improved (Figure 1E).

Synthetic biology is a field that uses engineering strategies, including computational models for biological investigation. An emergent area of synthetic biology is engineering and controlling microbial communities (Serrano, 2007; McCarty and Ledesma-Amaro, 2019). This can be done with genetic tools by creating synthetic microbial communities, those where species are developed or modified and introduced in the human microbiome (Habibi et al., 2012). For instance, bacterial strains can be designed to improve a certain pathway leading to increased production or consumption of certain metabolites leading to a specific function (Roell et al., 2019). This can act as a delivery system at a specific microbial community in the human microbiome. An exemplary previous study has shown that genetic circuits can reprogram the identity of cells in order to treat diabetes (Duan et al., 2015). Overall, this demonstrates the potential applications of synthetic biology in medicine and how it may deliver therapeutics (Figure 1F).

In this review, we will discuss the range of multi-omics data types and computational methods that are used in human microbiome research. We highlight possible outcomes for integrating synthetic biology and systems biology in health and disease applications. Finally, we discuss whether it is possible to integrate both fields in human microbiome studies.

## Technologies Used to Study the Human Microbiome

**FIGURE 2:**
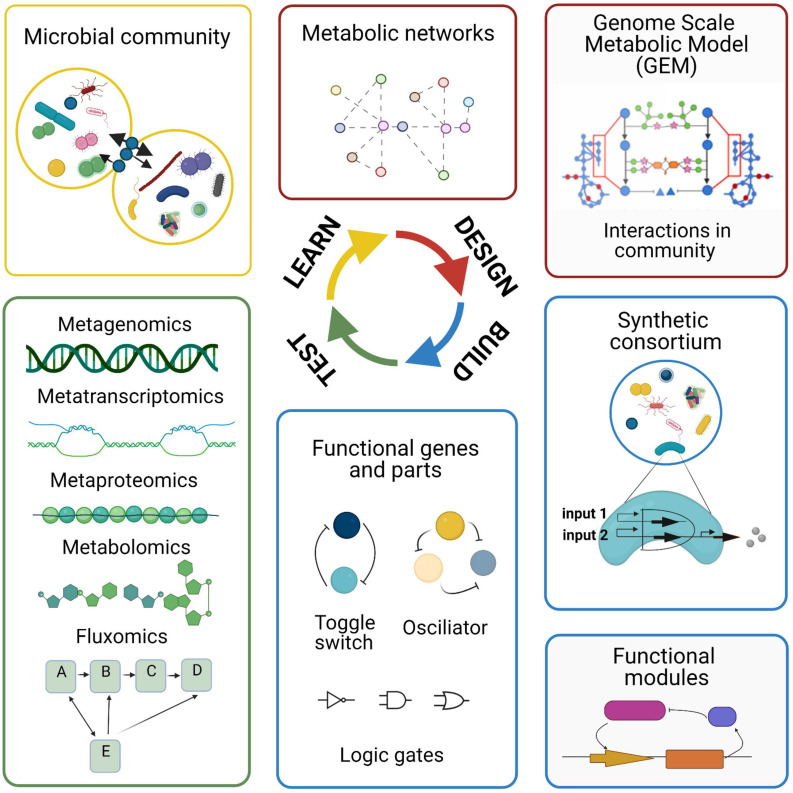
Advantages and limitations of omic technologies used in microbiome research. The different omic techniques allow for the measurement of different features (the genome, transcriptome, proteome, and metabolome) within a biological sample. After data generation, the data are processed and analyzed using bioinformatic approaches.

Microbiome research has benefited from recent advances in sequencing techniques and computational tools. There are different computational approaches used to study the microbial species of the human microbiome, to understand the microbial community composition and functions to unravel potential interactions. These methods include metagenomics, metatranscriptomics, metaproteomics, metabolomics, and single-cell omics. These individual methods alone provide limited mechanistic insight. However, combining these techniques with modeling approaches can help in elucidating interactions and predicting the behavior of the microbial community. Each omics technique has advantages and limitations when applied to study microbial communities (Figure 2).

### Metagenomics

A method commonly used to study microbiome composition is amplicon sequencing (also known as 16s rRNA sequencing). In this method, the DNA is extracted and a specific region of the 16S rRNA gene is amplified and sequenced, and the generated sequences are identified using a reference database (De Oliveira Martins et al., 2020). This method allows for the identification of a specific organism at different taxonomic level (Johnson et al., 2019) and the functional profiles of bacterial communities can be predicted by using different bioinformatic pipelines such as Tax4fun, PICRUSt2, and Vikodak (Aßhauer et al., 2015; Nagpal et al., 2016; Douglas et al., 2020). It is generally fast, cost-effective, and an appropriate tool to characterize unculturable bacterium. Although 16s rRNA sequencing is a powerful tool to study microbiome communities, there are limitations. For instance, this method has a limited taxonomic coverage and can identify bacteria and archaea but not viruses and fungi. In most cases, bacteria can only be identified at genus level due to the high similarity between 16s rRNA genes from closely related species (Osman et al., 2018).

Using amplicon sequencing has revealed the diverse character of the microbiome and advanced our understanding of its role in health and disease in multiple body sites (Grice et al., 2009; Guerrero-Preston et al., 2016). In addition, microbiome studies have shown that microbial profiles have little individuality at species level but high individual specificity on strain level (Oh et al., 2016; Abu-Ali et al., 2018). Hence, community profiling at a finer resolution is needed for a better understanding of the bacteria within a community.

Another technique used to study microbial communities is shotgun metagenomics. Shotgun metagenomics can reveal the compositional and functional information of the microbial communities. This technique analyzes abundance of microbial species and provides insights in the functional profile of the community (Cheng et al., 2019). Metagenomics studies have highlighted differences in composition of the microbiome and provide evidence that a dysbiosis in microbial communities play a role in the pathogenesis for diseases. For instance, several studies have shown that the strains of *E. coli* and *Ruminococcus gnavus* have been associated with inflammatory bowel disease (Joossens et al., 2011; Fang et al., 2018).

The general approach of shotgun metagenomics starts with fragmentation of DNA resulting in many short reads which are reconstructed into a consensus sequence and aligned to a reference genome to identify specific genes and functions (Lavezzo et al., 2016). An advantage of this method is that it allows for the detection of low abundant species of microbial communities (Truong et al., 2017). This method identifies the sequences of all organisms such as fungi and viruses, which cannot be detected by other sequencing methods. Novel organisms can be identified from a community in comparison with traditional culture-based techniques in which microbial organisms were isolated and individually studied (Qin et al., 2010). Despite the benefits of metagenomics, there are still several challenges associated with this method. Data obtained with metagenomics is often complex and large due to its multivariate structure making informatic analysis difficult. New software tools have been developed to analyze this complex metagenomics data, for example, MicrobiomeAnalyst. This tool enables researchers to perform numerous tasks such as metabolomic network visualization, community and functional profiling, and statistical analysis. Besides, MicrobiomeAnalyst and other tools like Metaviz and PUMA are statistical analysis and visualization tools that improve metagenomics data analysis (Dhariwal et al., 2017; Mitchell et al., 2018; Wagner et al., 2018).

Despite extensive culturing and sequencing, the microbial reference genome remains undefined and there is a need for well-characterized collection of reference genomes. Hence, a culture-independent and reference-free approach is possible by using a metagenome-assembled genome binning method (MAG). This is a computational approach in which sequence reads are assembled into contigs which are then binned into metagenome assembled genomes (MAGs) based on sequence coverage and tetranucleotide frequency. This method enables new insights into species and functions within uncharacterized bacterial communities (Almeida et al., 2019; Nayfach et al., 2019). However, there is still the issue of the incompleteness of the MAGs (Frioux et al., 2020).

### Metatranscriptomics

The functional profile of microbial communities can be explored with metatranscriptomics. This technique provides information about which genes are expressed in a specific microbial environment. Metatranscriptomics in combination with metagenomics data, which provides the information for gene and species abundances, enables characterization of microbial transcription. Therefore, an in-depth functional profile of a microbe can be acquired such as active metabolic pathways in different contexts (Franzosa et al., 2014; Bikel et al., 2015). A longitudinal metatranscriptomics study of IBD patients showed that the expression of two specific organisms was the reason for the expression of a certain pathway at different time points, which correlated with disease severity (Schirmer et al., 2018).

A typical workflow of metatranscriptomics consists of extraction of cellular RNA and conversion of it to cDNA for preparation of a sequencing library. Then, the obtained sequence reads will be mapped against a reference genome. This strategy is limited by the information present in the database of the reference genome (Shakya et al., 2019). Another strategy is *de novo* assembly of the reads into transcript contigs (assembled reads). The drawback of this method is that it is limited by the ability of software programs to correctly assemble the contigs from the short reads (Hrdlickova et al., 2017). Metatranscriptomics can detect at strain-level resolution, is less susceptible to amplification biases, and precise quantification of the activity of the organisms can be obtained (Marcelino et al., 2019). However, metatranscriptomics has its own challenges including the need to obtain sufficient and high-quality RNA. Another limitation is that the presence of mRNA is not always an indication for the presence of protein or protein activity (Guimaraes et al., 2014). Host RNA contamination can occur, depending on the sample, and can be problematic as it complicates downstream data analysis. Also, reporting species-specific gene expression within microbial communities requires a large amount of data (e.g., reads) as it is necessary to consider species abundances within the community (Abram, 2015; Marcelino et al., 2019; Chung et al., 2020).

While metatranscriptomics microbiome analysis holds promise in enhancing our understanding of the microbial communities, several challenges need to be overcome. Using a standard for RNA isolation, and sequence analysis can provide better integration of metatranscriptomics for microbiome research. As such, metatranscriptomics enables better elucidation of functional alterations of the microbiome in a given context and provides information of when a healthy microbiome converts toward a disease-driven configuration.

### Metaproteomics

Metaproteomics aims to study the whole microbial community by measuring the expressed collective proteins. This proteomic profiling method provides a direct measurement of the functional state of the microbial community. Metaproteomics analysis not only provides information about the function of microbial communities but also about the community dynamics and structure (Wilmes and Bond, 2006). Cellular proteins carry out most functions such as transport, catalysis of biochemical reactions, and maintenance of cell structure. These functions provide a picture of the cell phenotype at the molecular level. This understanding can give us a stable picture of microbial community function. Metaproteomic research has revealed remarkable discoveries on the activity and functional pathways of microbial communities. For instance, a metaproteomic study applied to IBD revealed that different protein modules at the mucosal-luminal interface are found between healthy subjects and IBD patients (Li et al., 2016). In general, a metaproteomic approach consists of extraction followed by purification of the protein. Proteins are digested (either chemically or enzymatically) into peptides which are then separated using a technique named liquid chromatography (LC) before mass spectrometry (MS). To identify the proteins, the experimental mass spectra are compared with theoretical mass spectra from a protein database (Hettich et al., 2013). The main advantage of this technique is the identification of proteins and the assignment to taxa providing a better understanding of host physiology. There are some challenges associated with this technique. For example, there are many peptides which are common to most bacterial species and therefore indistinguishable from each other which might result in false positives. Fortunately, in recent years, new software has been developed to handle the requirements of complex mass spectrometry data and reduced the rate of false identifications (Heyer et al., 2017). A drawback is that the interaction between function and taxonomy is difficult to analyze (Easterly et al., 2019). Identifying proteins from a complex microbial community composed of thousands of species is difficult due to the absence of complete genomic sequences of poorly characterized or uncultivated species. Overall, a complete database containing the collection of all known protein sequences is essential to identify the proteins of microbiome samples.

### Metabolomics

A method that gives a snapshot of the physiology of the cells is metabolomics. This method studies the substrates and products of metabolism and as such provides a functional readout of the cellular state (Fiehn, 2002). Metabolites are exchanged between several species in the microbial community and host, and play key roles in biology as signaling molecules, energy sources, and metabolic intermediates. Hence, the metabolome is the most direct indicator of health or dysbiosis of a specific body niche, making metabolomics a promising method for personalized medicine (Jacob et al., 2019).

Metabolomics experiments can take a targeted or untargeted approach. In targeted metabolomics, the identified metabolites are compared with a reference database of known metabolites. As the standard reference database for many metabolites is lacking, this can limit the number of compounds that can be detected (Cao et al., 2020). Moreover, many metabolites are similar across species making it difficult to discern the biological source in a study (Roberts et al., 2012). The untargeted approach, however, tends to find as many metabolites as possible and may not precisely quantify, in absolute terms, all measurable metabolites but may provide a relative quantification. It is therefore a powerful technique but there may be a bias present toward the most abundant metabolites (Schrimpe-Rutledge et al., 2016).

A general workflow for metabolomics starts with collection of a sample from which metabolites are extracted. Sample collection method and storage conditions play important roles in metabolomics and can lead to biases in the results. This has been reviewed previously in several articles (Deda et al., 2015; Chen et al., 2016). After sample collection and metabolite extraction, the metabolites are quantified with an analytical technique such as mass spectrometry (MS) or nuclear magnetic resonance (NMR) (Chen et al., 2019). These analytical techniques have their own advantages and disadvantages in regards to quantifying and identifying intracellular and extracellular metabolites and have been reviewed in more detail elsewhere (Aretz and Meierhofer, 2016). For instance, MS is a highly sensitive method and has a wide dynamic range to detect and quantify hundreds of metabolites in a single measurement. One limitation of this technique is its poor performance in analyzing large samples and non-volatile metabolites (e.g., alcohols, aldehydes, and ketones) greater than 700 amu (Rowan, 2011; Theodoridis et al., 2012). On the other hand, NMR is widely used for metabolomics as this platform is easily quantifiable and requires little to no chromatographic separation. In addition, this method requires minimal sample preparation. However, it has low analytical sensitivity (Emwas et al., 2019).

Metabolomics has been used in many human microbiome studies to investigate key metabolites and biological networks associated with host–microbiota interactions as it profiles the metabolites found in biofluids. For instance, a study by Gawron et al. (2019) used saliva to understand the pathological changes occurring in the oral cavity during the transition from health to chronic periodontitis and reported a change in eight metabolites. This study demonstrated that metabolomics can give insights in the metabolic status of the oral cavity in chronic periodontitis (Gawron et al., 2019). Urine has been used to study the dysbiosis that occurs in IBD. Williams et al. (2009) showed that a metabolite named hippurate had low levels in the urine of patients suffering from IBD which is interesting as hippurate levels have been shown to correlate with the presence of *Clostridia* in the gut. A recent large fecal metabolomics population study demonstrated that the fecal metabolome can be influenced by host phenotypes (i.e., age, sex, and obesity), gut microbial composition, and host genetic influences. This demonstrates the strong association between metabolic output and gut microbial structure (Zierer et al., 2018). Another study investigated the associations between the blood metabolome and gut microbial α-diversity (sample diversity) and found that 40 plasma metabolites could be used to predict gut microbiome diversity and that specific metabolites (e.g., TMAO) were also related to human health (Wilmanski et al., 2019).

### Single-Cell Omics

While meta-omics approaches have aided in the insight of host–microbiota interactions, they have not provided information at the level of the single microbial cell. Single-cell analysis aims to study the cell-to-cell variation within a population of cells; hence, providing insight into cell function and intercellular heterogeneity (Dhawan, 2019). To perform single cell analysis, individual cells are isolated using methods such as flow activated cell sorting (FACS), microfluidics, or serial dilution (Zeb et al., 2019). Remarkable advances in the field of single-cell analysis and in particular single-cell genomics and transcriptomics (sc-RNAseq) have been made. However, profiling the single-cell proteome and metabolome are in their infancy due to high diversity and large dynamic range of the cellular proteome and metabolome. In addition, there is a difficulty in the amplification step and single-cell data requires simultaneous profiling of large number of individual cells to overcome the noise in the data (Zenobi, 2013; Streets et al., 2014; Lin et al., 2019). Single cell nucleic acid analysis (DNA or RNA) have been used in microbiome studies (Xu and Zhao, 2018). The advantage of single cell analysis is that it allows low abundance species, which may not be detected by metagenomic sequencing, to be identified. In addition, the function of individual microbes within a community can be studied (Zenobi, 2013; Streets et al., 2014; Tolonen and Xavier, 2017). A limitation is that cell-sorting is time consuming. Amplification biases and environmental contamination is frequently observed during single cell sequencing (Lasken and McLean, 2014). The technical aspects of many single-cell omics approaches are available in other reviews (Gawad et al., 2016; Svensson et al., 2018). Single cell sequencing has been used in intestinal microbiome research and has led to novel findings such as the identification of specific gut microbial cells which use host-derived compounds and the quantification of taxon abundances of the gut microbiome (Berry et al., 2013; Props et al., 2017). A review of the current applications of single-cell omics in model organisms and in humans as well as the potential it has to improve diagnosis and treatment is available in an excellent article by Strzelecka et al. (2018).

## Integration of Multi-Omics Data for Human Microbiome Research

In the previous sections of this review, we have described approaches for understanding microbial diversity and microbial community composition, activity, and functionality. These omics technologies provide different ways to study microbial communities, but currently there is not a single approach that provides a complete picture of the complex interaction of these communities. Integrating multiple omic tools and analyses are needed for a deeper understanding of the members of a microbial community.

The main challenge is integration of multi-omics data to elucidate the complex interactions in the microbial community, host and environment, and also reveal the underlying mechanisms of the microbiome in a holistic way. There are various bioinformatic pipelines available for processing microbiome omics data. More in-depth information on which bioinformatic pipeline to use for different experimental designs of microbiome study is extensively reviewed elsewhere (Aguiar-Pulido et al., 2016; Heyer et al., 2017; D’Argenio, 2018). Each stage of a microbiome study from experimental design to data analysis can impact the results and biological interpretation. Hence, standardization is necessary at the data-analysis stage and will bring consistency and comparability to the microbiome field.

An example of an integrative multi-omics study was carried out by Heintz-Buschart et al. (2016), who characterized microbiome functions in patients with familial type 1 diabetes using different omics techniques. This study underpins the importance of integrating multi-omics analysis for host–microbiome interaction studies (Heintz-Buschart et al., 2016). Another study by Price also highlights the importance of meta-omics analysis. In their study, they showed how a complex set of metabolites can disrupt the microbiome and trigger inflammatory reactions during flares of inflammatory bowel disease (Lloyd-Price et al., 2019).

Systems biology, with its holistic view, can offer an integrative platform to link the multi-omics data and elucidate the complex interactions between the different components. This field combines experimental and computational methods observed in multi-omics data (metagenomics, metatranscriptomics, metaproteomics, and metabolomics) to identify the molecular mechanisms that occur within complex networks, representative of biological systems (Pinu et al., 2019). The main goal of systems biology is to study the complexity of biological networks by understanding the cellular and/or tissue interactions at a systems level, using mathematical models representing existing connection within a cell/and or tissue.

As large-scale omics data become more accessible, the integration of this data remains challenging due to the multiple types of data processing. Each individual omics discipline (e.g., genomics, transcriptomics, proteomics, and metabolomics) uses specific analytic tools and experiment designs, which makes it difficult to undertake comparisons or integrate the multiple omics datasets. Assuming that the data of the individual omics techniques are of high quality and well validated, different platforms can be used to integrate the data. Recently, Misra et al. (2018) reviewed different tools, databases, and approaches to integrate multi-omics data. For instance, the method ortholog two-way projection to latent structures (O2PLS) can be used to combine multiple sets of omics data, by reducing the feature space and without the need for *a priori* biological information (Löfstedt and Trygg, 2011). This method has been used, for example, in an asthma study in which data matrices from transcriptomics and metabolomics were combined with other assays (Reinke et al., 2018). Web-based platforms such as 3Omics and MetaboAnalyst enable the integration of different omics data and generate inter-omics correlation networks which aid data visualization (Kuo et al., 2013; Chong et al., 2018). Another approach for multi-omics data integration is with mathematical modeling.

## Genome-Scale Metabolic Models for Prediction of Function for Human Microbiome

Systems modeling is based on a well-defined understanding of the system (e.g., transcriptomics or metabolomics) that is being studied so that new experimental findings can be compared against the predicted models. For instance, the COBRA (Constraint-Based Reconstruction and Analysis) toolbox contains a function that integrates modeling of experimental molecular systems biology data and enables the prediction of, for instance, phenotypic properties at a genome scale (Heirendt et al., 2007). Mathematical models in biology are a useful platform for either the integration of omics data for new discoveries or to perform simulations to generate new hypotheses (Dahal et al., 2020). There are different types of mathematical modeling approaches such as differential equation models, dynamic models, and constraint-based stoichiometric models which provide insights into the functioning of the microbiome (Zomorrodi and Segrè, 2016).

Among the modeling approaches, genome-scale metabolic model (GEM) has generated interest in host and microbial research fields. GEMs incorporate lists of biochemical information from a target organism that are connected and encapsulate information on the stoichiometry, compartmentalization, reaction directionality, and their associations to genes and proteins. Therefore, these models can establish gene-protein-reaction links, which make them applicable to integrate different omics data such as transcriptomics, proteomics, metabolomics, and fluxomics to set up a genotype–phenotype (Gu et al., 2019). GEMs can be applied in constraint-based modeling, through consideration of specific objective functions such as growth or substrates, using sets of constraints (Gu et al., 2019). Depending on the type of constraints and objective function, different optimization algorithms such as linear programming can be used to determine the optimum solution. Using these applications, GEMs have successfully been applied to construct tissue/cell and microbial-specific models. The reconstruction of GEMs have been reviewed elsewhere, and the number of reconstructed GEMs increases each year (Feist et al., 2009; Bordel et al., 2010). The reconstruction of GEMs for species in a microbial community can be limited by the lack of availability of a genome for an unculturable microbe or missing functional annotations. There have been efforts to generate several models for gut microbes using the existing available known genome (Magnúsdóttir et al., 2017); however, still the challenge is the reconstruction of GEMs species without fully referenced genomes, as using the metagenome assembled genomes to draft models could result in several gaps or missing information (Mendoza et al., 2019).

**FIGURE 3:**
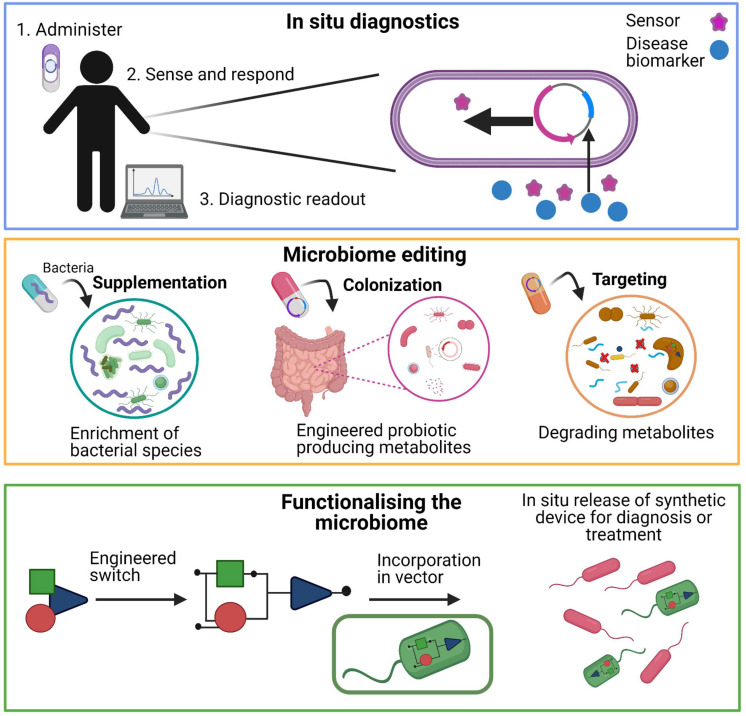
The design-build-test learn cycle (DBTL cycle) for integrating multi-omics data to infer detailed biological insight for designing new diagnostic and therapeutics for personalized and translational medicine. Different omics datasets can be generated from high-throughput studies and enables the characterization of cells and/or tissues in health and disease states. A combination of omics is necessary to reveal the complex behavior of cells and/or tissues that are present in different states. Integrating these high throughput omics data with systems biology methods such as genome-scale metabolic models can lead to more in depth biological knowledge revealing molecular mechanisms involved in health and disease states. Using synthetic biology, different engineering strategies can be employed to construct an organism that performs the desired function(s).

GEMs have been widely implemented in human microbiome studies to understand the interactions between the host and the microbiota as well as the effect of the microbiome composition on host. Diseases such as cancers, obesity, type 2 diabetes, and non-alcoholic fatty liver disease have been studied using context-specific GEMS (Shoaie et al., 2013; Ji and Nielsen, 2015; Mardinoglu et al., 2015; Shubham et al., 2017; Bidkhori et al., 2018; Rosario et al., 2018). GEMs can be constrained by using uptake/secretion reactions of metabolites, transcription data, rate of turnover of molecules through a metabolic pathway, also known as flux, and the gene expression state (on or off) based on information of high-throughput data (Bordbar et al., 2014). These constrains can specify a particular state or condition in which information of the overall metabolic capacity of the microbes can be obtained. Multiple GEMS can be joined together along their extracellular compartments to build a community model. This community model can be linked to a “common compartment” mimicking a certain body niche such as the human gut. This approach can be used to identify beneficial bacteria for human health and hence be used for the treatment of disorders that are associated with the human microbiota. Simulations such as flux variability analysis can estimate the flux span (minimum or maximum possible difference) for a specific metabolic exchange reaction of bacterial strains in a microbial community (Gudmundsson and Thiele, 2010). Also, pairwise analysis of microbes (can predict six different interactions: competition, parasitism, amensalism, neutralism, commensalism, and mutualism) in a community can be undertaken as well as measuring how the metabolic relationships change when introduced to different diets (Heinken et al., 2019). In the human gut microbiota, short-chain fatty acids (SCFAs) are bacterial metabolites produced in the colon and have been shown to impact on human health. With the help of GEMs, it was found which microbes produced specific SCFAs and that the concentration of SCFAs is low in Crohn’s disease (Bauer and Thiele, 2018). Thus, GEMs can give an insight into altered molecular processes in the development of diseases. Using GEMs and integrating high-throughput data gives us insights into microbial communities and is key to understanding the microbiome. This knowledge can directly or indirectly contribute (e.g., with biotechnological applications) to changing the microbiome to benefit the medical industry (Figure 3).

## Synthetic Biology: Potential Application of Diagnostics to Human Microbiome Research

Early stage diagnostics are essential to detect diseases in the initial phases, when treatments tend to be more effective. Diagnostic applications of the microbiome are needed to assess dysbiosis states or specific diseases. For most diagnostics, a sample from the patient is obtained (e.g., urine, saliva, feces). This sample is then studied in a clinical laboratory, providing information about the health of the patient. Using multi-omics techniques, applied to the human microbiome, resulted in the discovery of biomarkers, for example, in periodontal disease and oral cancer (Yoshizawa et al., 2013). Besides traditional diagnostics, new diagnostics using synthetic biological systems can be employed to develop devices that can sense a stimulus *in situ* and immediately provide a therapeutic.

**FIGURE 4:**
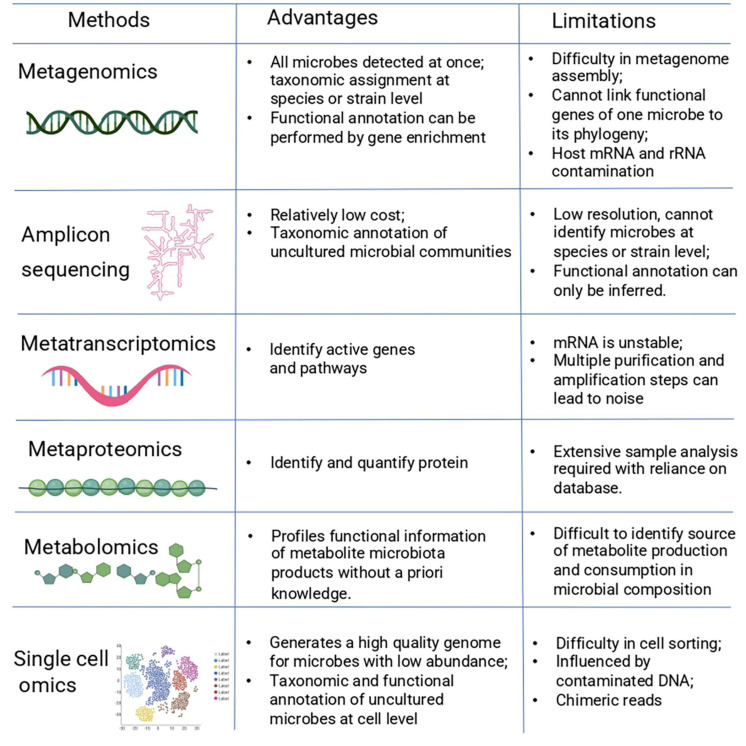
Potential application of diagnostics and therapeutics for human microbiome research. Top panel shows how synthetic biology enables the design of biosensors. This biosensor is a molecular module that can sense and process a biological signal which can be read by a diagnostic device. The middle panel shows different approaches of how the microbiome can be edited by supplementing the communities with certain bacteria that leads to enrichment of bacteria in the community. Colonization of the microbial community with engineered bacteria can lead to the production of certain compounds. Targeting specific bacterial species can selectively eliminate deleterious metabolites from the microbiota. The bottom panel show how a genetic circuit can be engineered and incorporated in a vector that can either diagnose and/or release the therapeutic treatment.

Diagnostics can be designed with bacteria engineered with the capacity to detect a signal with a high sensitivity and to integrate and respond to that signal with an appropriate output. These so-called biosensors can be used for example in the human intestinal tract in which they respond to perturbations in a dynamic environment. Biosensors generally consist of one or two component systems, which can respond to molecules such as cytokines (Zav’yalov et al., 1995), hormones (Clarke et al., 2006), temperature (Piraner et al., 2017), and metabolites (Pickard et al., 2014; Figure 4). Previous studies show how differently designed biosensors can sense environmental signals and regulate gene expression in *ex vivo* samples and murine models (Bonnet et al., 2013; Siuti et al., 2013; Mimee et al., 2015). Recently, a specific heme-sensitive probiotic biosensor was designed as a diagnostic tool to monitor gut health and to detect gastrointestinal bleeding. For this biosensor, two bacteria were used to sense blood in the extracellular environment and modify it to produce a bioluminescence output signal. This system was added in a gut-friendly bacterial strain, which could be ingested as a pill. This study shows great promise for detecting small molecules produced in the gut, which are difficult to detect using traditional diagnostics (Mimee et al., 2018). Another study by Riglar et al. (2017) showed how live, engineered bacteria could colonize the mouse gut for 6 months and monitor an inflammatory marker (tetrathionate) during the course of the disease (ulcerative colitis). This study demonstrates how a robust synthetic memory circuit is suitable for longitudinal studies and shows great promise in the development of stable engineered biosensor strains for *in vivo* studies (Riglar et al., 2017).

Another diagnostic approach to detect diseases is by using quorum sensing (QS) molecules. This is a mechanism by which bacteria communicate using extracellular chemical signaling molecules. QS has been tested *in vitro* in which bacteria detect a molecule and respond by producing another compound such as an antimicrobial peptide (AMP) (Saeidi et al., 2011; Hwang et al., 2014).

Quorum sensing can be used to control the expression of engineered functions and to restrict expression to a relevant body site. For instance, in a study by Swofford et al. (2015), the authors engineered the species *Salmonella enterica* to turn on gene expression in tumors, as there is a high cellular density, and remained off in other off-target locations such as the liver where a low bacterial cell density was present. This study shows promise in using quorum sensing mechanisms to control expression at a specific site. Also, autoinducer (AI-2) has been shown to be present in the human gastrointestinal tract and this molecule is produced by many gut bacterial species, which promote colonization of Firmicutes over Bacteroidetes and can limit *Vibrio cholerae* infections (Hurley and Bassler, 2017). Hence, QS provides an opportunity to intervene in gut dysbiosis.

## Synthetic Biology: Current and Potential Application of Therapeutics for Human Microbiome Research

The field of synthetic biology provides ways to rebalance the dysbiosis of the microbiome, therefore preventing the development or treating diseases. Recent advances in this field make use of genetically engineered bacteria to deliver targeted therapies for treatment of diseases such as HIV, inflammatory bowel disease, diabetes, and cancer (Duan et al., 2015; Gosmann et al., 2017; Ho et al., 2018; Charbonneau et al., 2020). This new approach has several advantages in comparison with the traditional therapeutics (Braat et al., 2006; Lagenaur et al., 2011; Takiishi et al., 2017; Zheng et al., 2017). First, the production costs of medication would decrease as the microorganisms will produce the therapeutic directly in the human body. Second, reduced side effects are expected as the administration is non-invasive and the therapeutics are administered locally.

There is a growing interest in microbiome engineering for shaping the microbiota. Currently, there are several strategies to manipulate the microbiota which can be classified as additive, subtractive, or modulatory. An additive therapy entails that specific strains or communities can be added to the host microbiota. These strains or communities can be natural or engineered microorganism. The subtractive therapy refers to a therapy by which specific strains need to be eliminated or the production of certain metabolite must be reduced to improve or cure a disease. A modulatory therapy involves probiotics or/and prebiotics which modulate the composition of the endogenous microbiome. This section will discuss how the microbiota can be altered to treat diseases (Figure 4).

A therapy that has gained lots of attention over the years is fecal microbiota transplantation (FMT). This method restores the gut microbiome by transplanting stool from a healthy donor into the gastrointestinal tract of the disease-associated microbiome. This transplantation has been successful in patients with *Clostridium difficile* infection (Kelly et al., 2014). As FMT relies on established microbial communities from healthy donors, a refined approach to transplantation can be obtained with designing synthetic communities. These synthetic communities could replicate the same functions as the natural communities which are present in healthy donors.

The first generation of microbiome therapies consisted of prebiotics and probiotics. Prebiotics are non-digestible foods that are degraded by the gut bacteria (Delbès et al., 2018). Most prebiotics consist of carbohydrates such as fructans, starch, and oligosaccharides. Fermentation of these prebiotics results in the production of SCFAs, which can have multiple effects on the human body (Sasaki et al., 2018). Previous studies show that diseases such as ulcerative colitis, Crohn’s disease, and IBD have a significant reduction in butyrate-producing bacteria (Cho and Blaser, 2012). As prebiotics promote the growth of certain bacterial species, this growth can be transient and limit the health benefits. Thus, to observe a significant effect, the prebiotics must be consumed regularly. Alternatively, probiotics are another way to alter the microbiota composition (Gibson et al., 2017). These are live microorganisms found in certain foods or supplements. Probiotics work by modulating the imbalance that is present by increasing certain bacterial species which have beneficial health effects. An example of a probiotic food is yogurt which contains the bacterial species *Streptococcus thermophilus* and *Lactobacillus bulgaricus* (Ejtahed et al., 2011). Probiotics and their treatment effects have been studied in an increasing number of clinical trials (McFarland, 2015). A meta-analysis reported that probiotic administration had a beneficial role on the metabolic profile of managing type 2 diabetes patients (Kocsis et al., 2020). Recently, a review by Dudek-Wicher et al. (2020) described the influence of probiotics on human health and summarized the known mechanism of actions of probiotics as well as the clinical trial results of different diseases. Despite the promising results about probiotics, they have not shown significance yet in clinical trials (Dudek-Wicher et al., 2020). This can be due to the gap in knowledge regarding the mechanisms by which probiotics modulate various functions. Also, drawing a conclusion based on used strains of probiotics for a disorder remains difficult as clinical trials use different doses and formulation (Brüssow, 2019). Other reasons include the difficulty of isolating bacteria but also due to the genetic differences across individuals (Lampe et al., 2013). Lastly, many trials deal with methodological problems or underpowered studies (Brüssow, 2019). However, understanding the limitations and the mechanisms by which disease are caused provides ways to design new probiotics which counteract the limitations. This can be done with engineering probiotics. For example, probiotics have been engineered so that bacteria produce chemicals or proteins. Steidler et al. (2000) used such an approach in which a bacterium produced the human interleukin-10 to reduce inflammation and reduced the disease colitis (Steidler et al., 2000).

More recently, the bacterium *Escherichia coli* Nissle 1917 has been used as a probiotic and engineered to detect the environmental signal, tetrathionate (molecule produced in the inflamed gut). This detection resulted into production of a microcin, capable of inhibiting the organism responsible for the inflammation (Palmer et al., 2018). Moreover, there are different companies that engineered probiotic bacteria for the treatment of various diseases. Xycrobe has developed an engineered bacteria able to penetrate the top layer of dead skin enabling a biotherapeutic to be delivered. With this approach, the skin microbiome is targeted, and the engineered bacteria are directly delivered to the target site treating acne (Cully, 2019). Another company, Osel Inc., focuses on the vaginal microbiome to treat bacterial vaginosis. They use the species *Lactobacillus* to maintain proper vaginal health. Moreover, the lactobacillus strain has been engineered in a way that inhibitors of HIV are produced resulting in protection against HIV (Clarke et al., 2006). Although engineering probiotics seems promising, more research is needed to use probiotics as a therapeutic agent.

Another attractive therapy to treat or improve dysbiosis is by using small molecules produced by microbes that modulate host physiology (Franzosa et al., 2019). Many metabolites serve as a mean to communicate between host and microbes. Targeting downstream signaling pathways of the microbiome leading to a dysregulation or an excess of certain metabolites can be used to treat certain diseases. For instance, a study by Sharon et al. (2019) showed how two metabolites named taurine and 5-aminovalerate were found in the stool from people with autism, and that when administered to a mouse model of ASD the behavioral symptoms improved (Sharon et al., 2019). Another literature study by Descamps et al. (2019) provides an overview of small molecules that are or can be used to treat microbiome-associated diseases (Descamps et al., 2019). Metabolite-based therapies are attractive for multiple reasons. First, they are suitable for different routes of administrations (Altaf-Ul-Amin et al., 2019). Second, they are generally stable and have a low toxicity (Smith and Obach, 2009). Limitations of metabolite-based therapeutics are the short half-life time and that some metabolites are highly cell-type specific. Thus, to use metabolites as therapeutics, the full characterization of different metabolites is needed to understand their action and their side effects. Currently, different biotech companies are exploring the use of small molecules and many are in phase I and II clinical trials to treat dysbiosis (Cully, 2019).

A different application which has gained attention is using bacteriophages. These small virus-like organisms consist of a protein capsule around an RNA or DNA genome and can infect a certain type of bacteria. A bacteriophage can be designed to target a specific bacterial strain in the microbiome and eliminate a potential pathogenic strain thus modulating the microbiome. This approach has the advantage of being target specific. Another approach is using phages as a delivery mechanism. For instance, the delivery of heterologous gene networks to target bacteria can disrupt their structure by expressing certain genes (Clark and March, 2006). In addition, the DNA editing tool CRISPR-Cas9 (Hamilton et al., 2019) can be delivered with phages so that designated strains can be removed. Different companies such as Eligo Biosciences, Locus Biosciences, and SNIPR-biome use CRISPR to protect or enhance the microbiome for precision medicine to treat certain conditions.

Besides phages, another way to alter the microbial community is with the help of AMPs. These are a diverse group of bioactive small proteins; key regulators of interaction between microbes and host. Especially the gastrointestinal tract has been explored extensively. For example, an *E. coli* strain has been modified to overproduce the compound arginine to lower the amount of blood ammonia from the intestine to correct for rare metabolic disorders. This engineered strain had no serious adverse events and is now in clinical trials (Kurtz et al., 2019). Also, *E. coli* has been engineered to produce multiple AMPs to specifically target and kill specific *Enterococcus* species to decrease murine colonization (Geldart et al., 2018).

## Limitations of Systems and Synthetic Biology and Future Opportunities

As described previously, synthetic and systems biology offer very promising opportunities to understand, diagnose, and treat microbiome-associated disorders. However, they still have several challenges. One of the major limitations in microbiome studies is recovering reliable assembled genomes form metagenomics studies with higher quality draft, therefore less accurate functional annotations to unravel the microbe’s specific phenotype. This can hinder the reconstruction of biological networks for incomplete genomes such as GEMs within large ecosystems. Advances in technologies such as culturomics could offer isolation of new strains and generating new genomes. Culturomics is a high-throughput culture approach and describes the microbial composition. In this method, various selective and/or enriched culture conditions are coupled to MALDI-TOF mass spectrometry and targeted sequencing (Greub, 2012). However, this technology is laborious, costly, and time consuming. Moreover, a deeper understanding of host physiology will make the construction of synthetic biology tools more reliable, precise, and robust. This will enable us to design microbial diagnostics and therapeutics to target human pathologies associated with human microbiome for which there is still an urgent unmet need.

Merging the fields of synthetic biology and human microbiome research comes as well with challenges. One technical issue is that most synthetic biology–based approaches have been tested *in vitro* or in murine models, and the performance within the human body still needs to be proven. Issues around the stability of some of these approaches, their half-life in the microbiome, and their colonization of specific target areas of the body need further development. Another matter of concern is the use of genetically modified organisms. In particular, the long-term effect of introducing altered species into the natural environment of the human body has not been well studied. In addition, recombinant organisms can transfer their genetic material to other microorganisms found in the human microbiome. This process is known as horizontal gene transfer and can lead to unintentional spread of modified DNA. However, engineered microbes can be constructed with a kill switch or with systems to eliminate the heterologous genetic circuit (Caliando and Voigt, 2015; Chan et al., 2016). This would limit the long-term colonization of genetically modified organisms. Microbiome engineering shows promising prospects in improving human health. However, its benefits need to be balanced against the risks. The safety and regulation of using natural and genetically engineered microbial strains is a widely discussed topic and the right frameworks need to be constructed before they can be used as new diagnostics or therapeutics (Prakash et al., 2011; Wong and Chu, 2013; Lindemann et al., 2016; Charbonneau et al., 2020).

There has been a great progress in the past decades in human microbiome research. Advancement in systems biology contributed to the newfound knowledge on the microbiome. This knowledge has demonstrated great success for understanding microbiome associated diseases. However, there is still a lot about the microbiome that is unknown. Especially, the dynamics and interactions of microbiomes are largely not understood. Studying the direct interaction between the host and microbiome remains a challenge. However, recently, microfluid systems called organs-on-a-chip were designed to mimic an organ *in vitro*. In this study, the human gut microbiota was co-cultured with intestinal epithelial cells and local immune cells providing an *in vitro* model in which the host–microbiome interactions could be studied (Jalili-Firoozinezhad et al., 2019). This method shows potential and opens new opportunities for personalized medicine and human microbiome studies. Engineering approaches will allow us to “communicate” with other microorganisms or the host. Together with omics technologies, this can help to unravel the microbiome remaining mysteries. In comparison with the gut, the microbiomes of the skin, oral cavity, genitals, and airways is in its infancy. Thus, more research needs to be directed in the aforementioned microbiomes. Synthetic biology not only provides tools to develop a deeper understanding of the microbiome but also has shown great promises toward diagnostics and therapeutics. Challenges of synthetic biology–based microbiome therapeutics and diagnostics are mostly associated with the incomplete knowledge of the microbe–microbe and host–microbe reactions. When this understanding is improved, it will go hand in hand with enhanced and localized diagnostics and therapeutics.

## Author Contributions

SS and RL-A conceived the project. RL-A supervised the work. BE, SS, and RL-A wrote and revised the manuscript. All authors contributed to the article.

## Conflict of Interest

The authors declare that the research was conducted in the absence of any commercial or financial relationships that could be construed as a potential conflict of interest.

## Publisher’s Note

All claims expressed in this article are solely those of the authors and do not necessarily represent those of their affiliated organizations, or those of the publisher, the editors and the reviewers. Any product that may be evaluated in this article, or claim that may be made by its manufacturer, is not guaranteed or endorsed by the publisher.
